# Supplementary material for: Semantic knowledge graph fusion for fake news detection: Unifying content-based features and evidence-based analysis in the COVID-19 infodemic
Source: PLoS One. 2025 Jul 1;20(7):e0321919. doi: 10.1371/journal.pone.0321919 (PMC12212873; doi:10.1371/journal.pone.0321919)
Supplement: Attachment — Submitted filename: response new pone.pdf [file pone.0321919.s001.pdf]

Response to Reviewers
Manuscript ID: PONE-D-23-32696
Title: *"Semantic Knowledge Graph Fusion for Fake News Detection: Unifying Content-based Features and Evidence-based Analysis in the COVID-19 Infodemic"*

Dear Dr. Asif Ekbal and Reviewers,

We sincerely appreciate the valuable feedback and suggestions provided by the reviewers. We have carefully considered each point and revised the manuscript accordingly. Below is a detailed response to the reviewers' comments.

Reviewer #1:

1. **Inclusion of more related work relevant to the domain:**
   - **Response:** We have expanded the Related Work section to include additional relevant literature that covers key areas.
2. **Generalization of the proposed method by evaluating on other datasets:**
   - **Response:** We acknowledge that evaluating on a single dataset limits the generalizability of our results. While we have not conducted additional experiments on other datasets, we appreciate the importance of this step. We plan to explore this in future work to validate our approach across different domains.
3. **Details of experimental setups and in-depth analysis:**
   - **Response:** We have provided additional details on the experimental setup, including:
     - Hyperparameter tuning and model configuration for the algorithms used.
     - The use of cross-validation to ensure robust results.
     - The number of training iterations and evaluation metrics (mean, standard deviation) for each model.
   - The revised Results and Discussion section now includes a thorough analysis of our experiments, with both qualitative and quantitative results.
4. **Hyperparameter tuning and model design choices:**
   - **Response:** We have added an explanation of the hyperparameter tuning process, including learning rates, batch sizes, and the number of epochs for each model. Additionally, we have discussed the design choices behind our model architecture,

explaining the rationale for using DistilBERT and BioBERT alongside Knowledge Graph integration.

5. **Reproducibility and availability of code:**
    - **Response:** We have made the code used in our experiments publicly available in a zenodo repository (https://doi.org/10.5281/zenodo.12669980) to ensure reproducibility. The code includes detailed instructions for running the experiments.
6. **Shallow conclusions:**
    - **Response:** The Conclusions section has been rewritten to reflect a deeper understanding of the findings. We have also expanded the discussion on future work, including the potential for real-time application of our model and how it can be extended to other domains of misinformation.

Reviewer #2:

1. **General Feedback:**
    - **Response:** We thank Reviewer #2 for their positive feedback on the significance of our research. The importance of developing a robust fake news detection model is critical, especially in the context of the COVID-19 pandemic, as highlighted by Reviewer #2. We have further improved the clarity and detail of our methodology, expanding on the role of Knowledge Graphs and transformer models in enhancing detection accuracy.

Additional Changes:

- **Spelling and Grammar:** A thorough spell check and grammatical review of the manuscript have been completed.
- **New Authors:** The contributions of additional co-authors, Muhammad Shahid Anwar, Patrik B¨ohm, Jaroslav Frnda, in the revision process have been appropriately acknowledged.

We hope these revisions address all concerns raised by the reviewers, and we believe the manuscript has been significantly strengthened as a result. We are grateful for the opportunity to resubmit and look forward to your feedback.

Sincerely,
Rayees Ahmad Dar
On behalf of all co-authors
