# Supplementary material for: Semantic knowledge graph fusion for fake news detection: Unifying content-based features and evidence-based analysis in the COVID-19 infodemic
Source: PLoS One. 2025 Jul 1;20(7):e0321919. doi: 10.1371/journal.pone.0321919 (PMC12212873; doi:10.1371/journal.pone.0321919)
Supplement: Attachment — Submitted filename: response_pone_r2.pdf [file pone.0321919.s002.pdf]

Rebuttal Letter to the Reviewers

Manuscript ID: [PONE-D-23-32696R1]
Title: "[Semantic Knowledge Graph Fusion for Fake News Detection: Unifying Content-based Features and Evidence-based Analysis in the COVID-19 Infodemic.]"
Date: February 1, 2025

Dear Editor and Reviewers,

We thank you for the time and effort you have invested in reviewing our manuscript. We have carefully considered all of the comments provided and have made significant revisions accordingly. Below we detail our responses to each of the reviewer comments.

Response to Reviewer #1:

Comment #1: Inclusion of Additional Related Literature:

Reviewer Comment: The reviewer noted that the manuscript had ignored several key works regarding:

- The effect of word embeddings on fake news detection,
- The use of transformer embeddings,
- The impact of document embeddings,
- Real-time architectures for fake news detection,
- Social network features, and
- The effect of multilingual transformers.

Response: We have significantly expanded the related work section in the revised manuscript (Subsections [Content based approaches] to include discussions of these topics. This new discussion provides a comprehensive review of literature related to the use of various embedding techniques, real-time architectures, and social network-based approaches, better situating our contribution within the current body of research.

Comment #2: Network Immunization Approaches:

Reviewer Comment: The reviewer observed that the manuscript did not discuss network immunization strategies, such as community detection methods, weighted directed spanning trees for real-time detection, and budget-based immunization algorithms.

Response: In response, we have added a new subsection[[network-immunization for fake news detection] in [related work] Section of the revised manuscript that reviews these network immunization strategies. This section explains how such approaches can be used to mitigate the spread of fake news, thereby enriching the context of our work.

Comment #3: Use of a Single Dataset:

Reviewer Comment: The reviewer pointed out that using only one dataset is insufficient for generalizing the conclusions and recommended additional experiments with at least two datasets.

Response: We appreciate the suggestion regarding the use of multiple datasets. However, due to the current scope and resource constraints, extending our experiments to include additional datasets is not feasible at this stage. To address this limitation, we have expanded the discussion section to analyze related work where multiple datasets have been employed and have clearly acknowledged this limitation in our study. We note that future work will include experiments on additional datasets to further validate our findings.

Comment #4: Exploratory Data Analysis (EDA):

Reviewer Comment: An in-depth exploratory data analysis was requested.

Response: We appreciate the reviewer's suggestion regarding exploratory data analysis (EDA). However, we would like to clarify that the primary focus of our study is on the development and evaluation of the CogiGraph framework for semantic and factual knowledge fusion in fake news detection. The dataset we utilized is already well-structured, balanced, and widely used within the community. As such, detailed EDA was not the primary objective and was deemed out of scope for this particular research.

Nonetheless, we have ensured a thorough description of the dataset's characteristics, including class distributions, source details, and data splits, in the manuscript's methodology section to provide transparency about the data used. We hope this clarifies our approach and justification for not conducting extensive EDA in this study.

Comment #5: Explanation of Evaluation Metrics:

Reviewer Comment: The reviewer requested detailed explanations regarding the selection of evaluation metrics.

Response: We sincerely appreciate the reviewer's valuable feedback regarding performance metrics. In the updated manuscript, we have comprehensively elaborated on the rationale behind the selection of evaluation metrics in the *Performance Comparison* subsection. Specifically, we have highlighted the importance of accuracy, precision, recall, and F1-score for a holistic evaluation of classification performance.

Each metric has been justified based on its ability to capture distinct aspects of the detection task. For instance, while accuracy provides a general assessment of correctness, it may not be suitable for imbalanced datasets. Precision and recall are emphasized as critical for understanding the system's effectiveness in identifying fake news without raising false alarms. The F1-score, being the harmonic mean of precision and recall, offers a balanced view when trade-offs exist.

Moreover, we acknowledge recent advancements in evaluation methodologies for misinformation detection and discuss their relevance, including considerations for imbalanced datasets and specific detection contexts. This enhancement aims to ensure that the metric selection is both comprehensive and aligned with the study's objectives. We trust that these additions address the reviewer's concern and improve the manuscript's clarity.

Comment #6: Conclusions, Study Limitations, and Proofreading:

Reviewer Comment:

> "The conclusions continue to be very shallow. What are the limitations of this study? Please proof-read and spell-check the article before resubmitting."

Response: We thank the reviewer for these important suggestions. In response:

- **Expanded Conclusions:** We have revised the conclusions to provide a more in-depth synthesis of our findings, discuss the broader implications, and outline potential directions for future research.
- **Study Limitations:** A dedicated Limitations subsection has been added in the Conclusion Section that explicitly outlines the limitations of our study, including our reliance on a single dataset and other potential factors affecting generalizability.
- **Proofreading and Spell-checking:** The manuscript has been thoroughly proofread and spell-checked to ensure clarity and readability.

Response to Reviewer #3:

Comment #1: Overall Comments:

Reviewer Comment: Reviewer #3 noted that all previous comments have been addressed and that the revised manuscript provides comprehensive details on the methodology, experiments, and findings.

Response: We thank Reviewer #3 for the positive feedback and are pleased that our revisions have met the expectations.

Response to Data Availability Comment:

Reviewer Comment: Have the authors made all data underlying the findings in their manuscript fully available?

Response: As mentioned in our previous response during Revision 1, we have made the code used in our experiments publicly available in a Zenodo repository: `https://doi.org/10.5281/zenodo.12669980`. This repository includes detailed instructions for replicating the experiments, ensuring full reproducibility of our research findings.

Furthermore, the dataset used for this research is publicly accessible from its original source as detailed in the manuscript. We believe these steps adequately address concerns related to data sharing and reproducibility.

Additional Changes:
In addition to the revisions detailed above, we have updated various sections throughout the manuscript to improve clarity and organization. We trust that these changes have significantly strengthened the manuscript.

We hope that the revisions and our responses satisfactorily address all concerns raised by the reviewers. We look forward to your positive consideration for publication.

Sincerely,

Rayees Ahmad Dar
University of Kashmir
rayees.csscholar@kashmiruniversity.net
